# ENGAGING STAKEHOLDERS TO ADDRESS ANXIETY IN LATINO/A CAREGIVERS OF OLDER ADULTS WITH CANCER

**DOI:** 10.1093/geroni/igae098.0854

**Published:** 2024-12-31

**Authors:** Kelly McConnell, Jaime Gilliland, Andrew Salner

**Affiliations:** Memorial Sloan Kettering Cancer Center, New York, New York, United States; Memorial Sloan Kettering Cancer Center, New York, New York, United States; Hartford HealthCare Cancer Institute at Hartford Hospital, Hartford, Connecticut, United States

## Abstract

Approximately two-thirds of the 2.8 million U.S. cancer caregivers care for an older adult and spend an average of 32.9 hours per week providing care. In this difficult context, over one-third of cancer caregivers report clinically significant anxiety that is often more severe than patient anxiety. Cognitive behavior therapy (CBT) is efficacious and considered a first-line anxiety treatment. However, CBT was developed and tested primarily in white populations. In 2019, Latino/a individuals made up 18% of the U.S. population with 60.6 million individuals. Approximately 149,000 Latino/a individuals are diagnosed with cancer each year, making cancer the leading cause of death in this population. Latino/a cancer caregivers report significant distress and reduced quality of life following the patient’s diagnosis. Yet, few psychosocial interventions have been developed for Latino/a caregivers. The purpose of this project was to develop a culturally competent CBT intervention for anxiety in Latino/a caregivers of older adults with cancer. Latino/a older adults with cancer (n=7) participated in focus groups to provide feedback on an initial version of the intervention. Qualitative analyses identified multiple themes including the role of faith in coping with cancer, the importance of social support in managing anxiety, and the benefits of connecting with others who are experiencing cancer. These themes provide insight into the mental health needs of Latino/a caregivers of older adults with cancer and informed modifications to the intervention. The feasibility, acceptability, and efficacy of the intervention will be examined in future clinical trials.

